# Increasing toll-like receptor 2 on astrocytes induced by Schwann cell-derived exosomes promotes recovery by inhibiting CSPGs deposition after spinal cord injury

**DOI:** 10.1186/s12974-021-02215-x

**Published:** 2021-08-09

**Authors:** Dayu Pan, Yongjin Li, Fuhan Yang, Zenghui Lv, Shibo Zhu, Yixin Shao, Ying Huang, Guangzhi Ning, Shiqing Feng

**Affiliations:** 1 Department, of Orthopedics, Tianjin Medical University General Hospital, Heping District, Tianjin, 300052 People’s Republic of China; 2 International Science and Technology Cooperation Base of Spinal Cord Injury, Tianjin Key Laboratory of Spine and Spinal Cord Injury, Department of Orthopedics, Tianjin Medical University General Hospital, Tianjin, People’s Republic of China; 3 Department of Urology, Shanghai Tenth People’s Hospital, Tongji University, Shanghai, 200072 People’s Republic of China; 4 Department of Neuroscience, Johns Hopkins University School of Medicine, Baltimore, MD USA

**Keywords:** Schwann cell, Exosomes, Axonal regeneration, Spinal cord injury, Toll-like receptor 2, Astrocyte, CSPG (chondroitin sulfate proteoglycan)

## Abstract

**Background:**

Traumatic spinal cord injury (SCI) is a severely disabling disease that leads to loss of sensation, motor, and autonomic function. As exosomes have great potential in diagnosis, prognosis, and treatment of SCI because of their ability to easily cross the blood–brain barrier, the function of Schwann cell-derived exosomes (SCDEs) is still largely unknown.

**Methods:**

A T10 spinal cord contusion was established in adult female mice. SCDEs were injected into the tail veins of mice three times a week for 4 weeks after the induction of SCI, and the control group was injected with PBS. High-resolution transmission electron microscope and western blot were used to characterize the SCDEs. Toll-like receptor 2 (TLR2) expression on astrocytes, chondroitin sulfate proteoglycans (CSPGs) deposition and neurological function recovery were measured in the spinal cord tissues of each group by immunofluorescence staining of TLR2, GFAP, CS56, 5-HT, and β-III-tublin, respectively. TLR2^f/f^ mice were crossed to the GFAP-Cre strain to generate astrocyte specific TLR2 knockout mice (TLR2^−/−^). Finally, western blot analysis was used to determine the expression of signaling proteins and IKKβ inhibitor SC-514 was used to validate the involved signaling pathway.

**Results:**

Here, we found that TLR2 increased significantly on astrocytes post-SCI. SCDEs treatment can promote functional recovery and induce the expression of TLR2 on astrocytes accompanied with decreased CSPGs deposition. The specific knockout of TLR2 on astrocytes abolished the decreasing CSPGs deposition and neurological functional recovery post-SCI. In addition, the signaling pathway of NF-κB/PI3K involved in the TLR2 activation was validated by western blot. Furthermore, IKKβ inhibitor SC-514 was also used to validate this signaling pathway.

**Conclusion:**

Thus, our results uncovered that SCDEs can promote functional recovery of mice post-SCI by decreasing the CSPGs deposition via increasing the TLR2 expression on astrocytes through NF-κB/PI3K signaling pathway.

**Supplementary Information:**

The online version contains supplementary material available at 10.1186/s12974-021-02215-x.

## Background

SCI was devastating disaster for patients and society. The World Health Organization (WHO) approximates that between 250,000 and 500,000 people suffer from a SCI each year [1, 2]. Over time, the lesion remodels and is composed of cystic cavitation and scar formation, both of which potently inhibit axon regeneration and neurons survival [2]. Among the diverse molecules which can partially facilitate or inhibit axon growth after SCI [3–6], CSPGs, which are highly upregulated after SCI and mainly produced by reactive astrocytes [7], can strongly inhibit axon growth by forming a non-permissive perineuronal nets with other extracellular matrix molecules [8–11]. However, the intrinsic mechanism of CSPGs attenuate neurons survival was still not well known.

TLRs are transmembrane proteins that play a critical role in pattern recognition receptors. They are expressed by macrophages, microglia [12–15], astrocytes [16], Schwann cells [17] and neurons [18]. It is well known that axon regeneration depends on the microenvironment that is favorable for regeneration [19]. Some findings indicate that TLR2 knockout will delay recruitment/activation of macrophages after sciatic neuropathy, reduce myelin debris removal efficiency and inhibit axon regeneration and movement recovery [20–23]. Therefore, we speculate that TLR2 activation on astrocytes may be related to the release of CSPGs and the regulation of axon regeneration. Furthermore, the expression of TLR2 could be triggered by Schwann cells (SCs) [24].

In the peripheral nervous system, SCs can promote dedifferentiation and proliferation of axons after injury, on the other hand, remove myelin and axon fragments. In parallel, the exosomes secreted by SCs have been applied to repair damage within the central nervous system [25–27]. The extracellular vesicles with exosome diameters of 10–150 nm [28–31] consist of a phospholipid bilayer membrane and contain RNA, proteins and lipids. They transported the cargo from the parent cell to the target recipient cell, where it was internalized and processed its contents [32]. Considering some recent studies have found that SCDEs can not only support axonal maintenance and regeneration after peripheral nervous system (PNS), but also contained many proteins closely related to axon regeneration and inflammation inhibition in central nervous system (CNS) [33], the SCDEs have been a novelty therapeutic option for the treatment of spinal cord injury.

In the present study, we systematically analyzed the effects of SCDEs on TLR2 on astrocytes involved in CSPGs releasing, neurons survival, and motor functional recovery after SCI. We demonstrated SCDEs could induce the expression of TLR2 on astrocytes, which lead to decreased secretion of CSPGs through NF-κB/PI3K pathway along with the improved functional recovery. The knockout (KO) of TLR2 on astrocytes was used to validate this finding. We believe that these results will inspire the investigation of SCDEs based on TLR2 and provide a novelty therapeutic option for clinical use that rescue the SCI.

## Methods

### Mice

All animal experimental protocols were approved by the Animal Care and Use Committee of Tianjin Medical University and Animal Ethical and Welfare Committee (AEWC) (Approval number: IRM-DWLL-2020120). All female mice used in different groups were housed in identical environments (temperature 22 °C–24 °C; humidity 60–80%) on a 12-h light–dark cycle (*n* = 90, at least 5 mice per group). We purchased C57BL/6 J (WT) female mice from Charles River. The GFAP-Cre (stock no. 024098) and TLR2^flox/flox^ (stock no. 004650) mouse strain were purchased from Jackson Laboratory. Heterozygous GFAP-Cre mice were crossed with TLR2^flox/flox^ mice. The offspring were intercrossed to generate the following genotypes: WT, GFAP-Cre (mice expressing Cre recombinase driven by GFAP promoter), TLR2^flox/flox^ (mice homozygous for TLR2 flox allele, referred to as “TLR2^f/f^” in the text), and GFAP-Cre; TLR2^flox/flox^ (conditional deletion of TLR2 in GFAP lineage cells, referred to as “TLR2^−/−^” in the text).

### Surgical procedures and treatment

We anesthetized the mice at 8 weeks of age with ketamine and xylazine by intraperitoneal (i.p.) injection. Laminectomy of a single vertebra was performed and severe crush spinal cord injury were made at the level of T10 using to expose the spinal cord. For severe spinal cord injury, a micro-mosquito curved hemostatic forceps (MEDLINE, MDS1222310) on first tooth and with a tip width of 0.2 inches were used to completely compress the entire spinal cord laterally from both sides for 15 s [33]. Mice in the sham group were subjected to laminectomy without crush. Bladders were manually massaged twice a day, antibiotic (Gentamycin sulfate, Abcam, ab146573) was administered once a day for 3 days post-surgery, analgesic (analgesic sodium, Abcam, ab145848) were received prior to wound closure and every 12 h for at least 48 h post-surgery. Animals were randomly assigned numbers and evaluated thereafter blind to genotype and experimental condition.

### Evaluation of the locomotive function

The locomotive function of hindlimb was evaluated at 1 day, 3 days, 5 days, 7 days, 14 days, 28 days, 42 days, and 56 days post-SCI using the Basso Mouse Scale (BMS) [34]. Automated gait analysis was also performed pre-surgery and 1, 2, 4, and 8 weeks post-surgery by using a “CatWalk” system (Noldus) [35]. All experiments were performed during the same period of the day (1:00 PM to 4:00 PM). For BMS, at least two examiners were blinded to the experimental group observed each mouse for 5 min. For Catwalk test, each mouse was trained to cross the Catwalk walkway daily for 7 days before SCI or control operation.

## Schwann cell isolation and cell culture

Primary Schwann cells were extracted from the sciatic nerves of adult WT female mice. In brief, after anesthetized the mice at 8 weeks of age with ketamine and xylazine by intraperitoneal (i.p.) injection, the sciatic nerves were extracted and washed three times with ice-cold phosphate-buffered saline (PBS) supplemented with 2% penicillin/streptomycin. Then, the surrounding membranes, muscular tissue, and epineurium were carefully removed by a stereomicroscope or fine forceps in a cell culture plate containing DMEM (Gibco) with 10% fetal bovine serum (FBS) and 1% penicillin/streptomycin. Subsequently, the nerves were cut into pieces 1 mm in length and digested in 0.05% collagenase-A (Roche, Germany) solution for 2 h at 37 °C. Three hundred microliters of fetal bovine serum (FBS) were added to halt enzymatic activity and centrifuged the mixture at 1500 rpm for 5 min. The cell was counted by using a hemocytometer and 1.2–2 × 10^4^ cells were cultured in poly-L-lysine-coated (Sigma, USA) plate containing DMEM (Gibco) with 10% FBS and 1% penicillin/streptomycin at 37 °C with 5% CO_2_. The medium condition was changed when the cells grew up to 80% area of the culture dish. The isolated Schwann cells were characterized by co-staining p75 and S100 (Supplementary Figure 1). For exosomes collections, Schwann cells were cultured in DMEM supplemented with 10% exosome-free FBS and 1 × penicillin/streptomycin (500 units of penicillin and 500 µg of streptomycin, Gibco Laboratories, Gaithersburg, MD) at 37 °C with 5% CO_2_, which was obtained by centrifuging FBS at 100,000 × *g* for 16 h at 4 °C.

### Isolation and culture of spinal cord astrocytes

We processed spinal cord astrocytes isolation and culture as described previously [36]. Briefly, the culture medium containing 10% fetal bovine serum (Gibco) and 1 × penicillin–streptomycin solution in DMEM (Gibco) were prepared. Six to 8-week-old mice were used for spinal cord astrocytes isolation and after, the mice with ketamine and xylazine were anesthetized. The meninges after taking the spinal cord out were removed and the tissue finely using a scalpel or razor blade to generate a tissue slurry was chopped. Then, the slurry to a 50-ml tube containing 5 ml of 4 mg/ml Papain (P3125, Millipore Sigma) in DMEM was transferred. Incubation was done in a 37 °C incubator with gentle shaking to ensure that the spinal cord tissue remains suspended for a minimum of 2 h. After that, centrifugation was done at 2000 rpm for 3 min and then the medium was aspirated and 5 ml of culture medium was added. After triturating the new medium with Pasteur pipette for at least 10 times, 10 ml of culture medium was added and the solution through a 30-μm nitex mesh filter was filtered. The solution at 1500 rpm was centrifuged for 5 min. Aspirate off the medium and the cells in culture medium up to 6 ml was resuspended. Then, carefully, the 6-ml cell suspension was added to the top of a gradient of Optiprep (D1556, Millipore Sigma) in a 15-ml centrifuge tube. Centrifugation was done for 15 min at 2200 rpm at room temperature and the debris from the densest band and above was discarded. Then, the cells and solution from the center to culture medium was collected. Centrifugation for 5 min at 2000 rpm was done, the supernatant was removed, and the cells in culture medium was resuspended. The cells was plated in a T75 flask that has been previously coated with poly-L-lysine and was incubated overnight in an incubator at 37 °C. Next day, the flasks were placed in a 37 °C shaker and were shaken overnight at 200 rpm to remove loosely adherent neurons and glia. After the overnight shake, the adherent cells in the flask were washed extensively with fresh culture medium and the residual cells (astrocytes) were fed with fresh media. At last, the adherent astrocytes were allowed to recover and expand over the course of 10–14 days, changing the media every 3–4 days before beginning each experiment. The isolated astrocytes were characterized by staining marker GFAP (Supplementary Figure 3). For the construction of an oxidative stress model that emulates SCI in vitro, astrocytes were incubated for 24 h in fresh medium containing 200 μM of H_2_O_2_.

### Preparation and purification of SCDEs

The resulting culture medium of Schwann cells was harvested to obtain exosomes by multiple ultracentrifugation; the first centrifugation was at 1000 × *g* for 10 min, while the second centrifugation was at 10,000 × *g* for 30 min. Subsequently, the supernatant was collected for the third time and ultracentrifuged at 100,000 × *g* for 1 h to precipitate exosomes (Beckman Optimal-100 XP, Beckman Coulter, Germany). After washing the SCDEs with PBS, the SCDEs were obtained after the final ultracentrifugation for 1 h at 100,000 × *g*. The SCDEs pellets were dissolved in Dulbecco’s phosphate-buffered saline (DPBS) and the total protein concentration of exosomes was quantified by the Bradford assay (Sangon Biotech, China).

### Characterization of SCDEs and injections

The morphology of the SCDEs was visualized using a high-resolution transmission electron microscope (TEM, Hitachi HT7700, Tokyo, Japan). Briefly, re-suspended SCDEs and mixed them with an equal volume of 4% paraformaldehyde (PFA) then adsorbed the mixture onto a glow-discharged, carbon-coated formvar film, which was attached to a metal specimen grid. Next, the grid was immersed with a small drop (50 μL) of 1% glutaraldehyde for 5 min then transferred to 50 μL uranyl-oxalate solution (pH 7.0) for 5 min after washed 8 times by 100 μL still water for 2 min each time. Subsequently, the grid was then transferred to 50 μL methyl cellulose-uranyl acetate (100 μL 4% uranyl acetate and 900 μL 2% methyl cellulose) for 10 min on ice. The sample was dried and examined in the TEM. Twenty-five microliters SCDEs per time (0.1 μg/μL) were suspended in 100 µl of DPBS and were injected into the tail veins of mice three times a week for 4 weeks since the induction day of SCI. Vehicle-treated mice received an equal volume of DPBS injected into their tail veins.

### Immunofluorescent staining

After anesthetized the mice mentioned before, mice sacrificed at different time points (1, 3, 5, 7, 14, 28, 42, and 56 days postoperatively) were first perfused with pre-cooled PBS via the ascending aorta to allow rapid and sufficient draining of blood flow, then perfused with 10% formalin solution for fixation. The following day, tissue was dehydrated in 30% sucrose at 4 °C for 48 h. For tissue embedding, spinal cords were embedded in optical cutting temperature (OCT) (Fisher Scientific, Cat#4583) and then been cut into 1 cm blocks centered on the lesion site. Spinal cord tissue was sectioned sagittally into 20-μm-thick serial sections and stored at – 20 °C.

The 20th–25th slices were selected and baked at 37 °C for 1 h, rehydrated with PBS for 5 min and then blocked at room temperature by blocking solution (0.5% Triton X-100 in PBS and 5% donkey serum) for 1 h. Next, the slices were incubated by primary antibodies at 4 °C overnight. The primary antibodies used were as follows: rabbit anti-5-HT (1:50, sc-65495, Santa Cruz Biotechnology), chicken anti-GFAP (1:500, ab4674, Abcam), rabbit anti-TLR2 (1:200, 12276S, Cell Signaling Technology), mouse anti-CS56 (1;250, ab11570, Abcam), rabbit anti-PGP9.5 (1:250, ab108986, Abcam), rabbit anti-Collagen III (1:100, ab7778, Abcam), mouse anti-Fibronectin (1:100, ab253288, Abcam), rabbit anti-GFAP (1;500, ab4674, Abcam), rabbit anti-p75 (1:50, ab52987, Abcam), and rabbit anti-S100 (1:50, ab34686, Abcam). The corresponding secondary antibodies were incubated 1 h at room temperature.

### Quantification analysis

Five alternate sagittal sections per animals were immunostained for 5-HT, GFAP, CS56, PGP9.5, TLR2, and DAPI. The zone spanning the lesion center and 3.6 mm in length, 2.8 mm in width apart was selected for analysis. The lesion site was photographed using a Zeiss LSM 780 confocal microscope. Measurements were carried out using the ImageJ/Fiji software. The nerve gap, which was determined using the ImageJ/Fiji software, was the distance between the rostral and caudal of PGP9.5-positive nerve in the injury area. And the 5-HT^+^ axon dieback was thresholded from middle of lesion to edge of 5-HT labeling. The CSPGs area occupied by CS56 signal was thresholded and determined by ImageJ/Fiji software.

### Western blotting

The SCDEs and cell pellets were lysed with radioimmunoprecipitation assay (RIPA) buffer. Load 30 µg of total protein per well and the proteins were separated by SDS-PAGE and blotted on a polyvinylidene difluoride (PVDF) membrane (Bio-Rad Laboratories, Hercules, CA, USA). The membrane was blocked by 5% bovine serum albumin (BSA) at room temperature for 1 h and then the primary antibody was incubated overnight at 4 °C. The primary antibodies used were as followed: rabbit anti-CD9 (1:2000; Abcam), rat anti-Alix (1:1000; Cell Signaling Technology), rabbit anti-CD63 (1:200, Santa Cruz, USA), rabbit anti-glyceraldehyde 3-phosphate dehydrogenase (GAPDH) (1:1000, Cell Signaling Technology, USA), rabbit anti-TLR2 (1:1000, Cell Signaling Technology, USA), rabbit anti-NF-κB p65 (1:1000, Cell Signaling Technology, USA), rabbit anti-Phospho-PI3 Kinase (1:1000, Cell Signaling Technology, USA), and rabbit anti-PI3 Kinase (1:1000, Cell Signaling Technology, USA). The protein bands were visualized by ECL substrate (ThermoFisher Scientific) and exposed under ChemiDoc XRS System (BioRad, USA).

### Statistical analysis

Each experiment was performed at least three times, and the results were expressed as means ± standard deviations (SD). For the data analysis, either one-way and two-way analysis of variance (ANOVA) followed by the Tukey’s multiple-comparison post hoc test or two-sample *t* test were performed with SPSS ver.13.0 (IBM SPSS, USA). Significant differences of behavioral analysis were used by repeated measures two-way ANOVA with Tukey’s post hoc test. The levels of significant difference among groups were defined and noted as *p < 0.05, **p < 0.01, ***P < 0.001, ****P < 0.0001.

## Results

### Characterizations of SCDEs

**Fig. 1:**
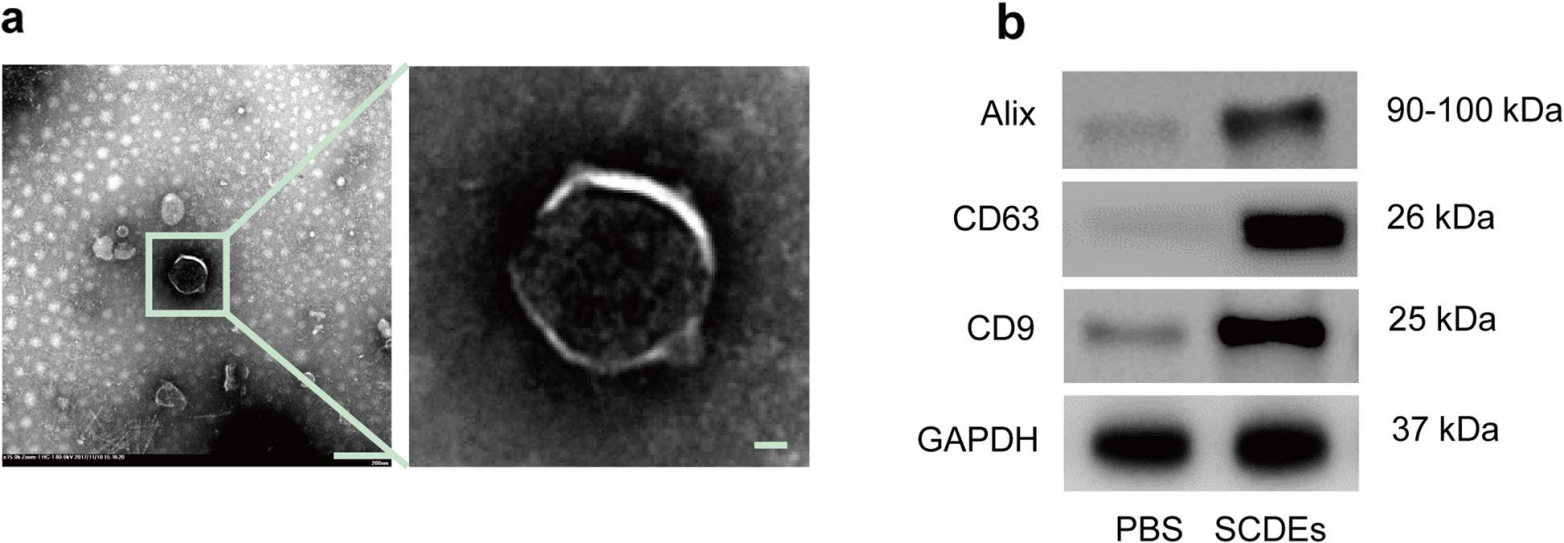
Characterizations of SCDEs. **a** Image of SCDEs under transmission electron microscopy (TEM). Scale bars denote the following: left side, 200 μm; right side, 50 μm. **b** Western blotting analysis of the expression levels of CD9, CD63, and Alix in SCDEs

To characterize the SCDEs extracted from primary cultured Schwann cells of mouse, we examined three markers which were expressed on exosomes: CD9, CD63, and Alix. Furthermore, the size of SCDEs ranged from 40 to 100 nm, as determined via TEM, offered additional evidence confirming that we had isolated SCDEs (Fig. 1a, b).

### SCDEs mediates the expression of TLR2 and the release of CSPGs

**Fig. 2:**
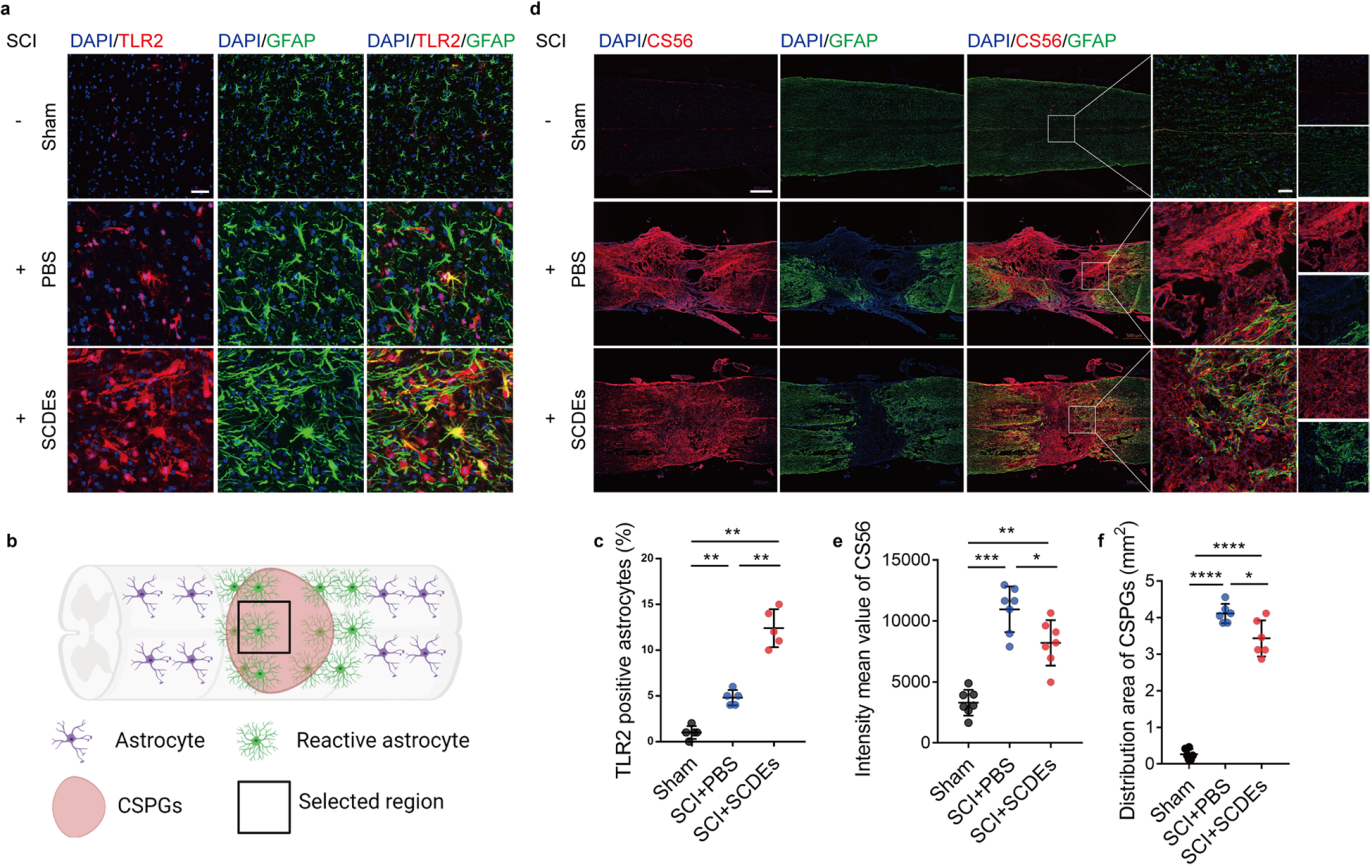
SCDEs mediates the expression of TLR2 and the release of CSPGs. **a** Representative images of immunofluorescent analysis of TLR2 (red), astrocyte marker GFAP (green), and DAPI (blue) staining of nuclei in spinal cord lesion site of T10 in sham group, PBS group and SCDEs group at 2 weeks after SCI. Scale bars = 50 μm. **b** A schematic diagram showing where the images in Fig. 2 were taken from. **c** Quantitative analysis of the TLR2-positive astrocytes (**P < 0.01, *n* = 5). **d** Representative images of immunofluorescent analysis of CSPGs marker CS56 (red), GFAP (green), and DAPI (blue) in different groups at 2 weeks after SCI. Scale bars = 500 μm. Right images are high-resolution versions of the boxed regions in the left images, scale bars = 50 µm. **e**, **f** Quantitative analysis of the intensity value of CS56 and CSPGs area (*P < 0.05, **P < 0.01, ***P < 0.001, ****P < 0.0001, *n* ≥ 6)

As astrocytes express TLR2 and TLR2 could subsequently activated astrocyte. To elucidate the relationship between TLR2 on astrocytes and SCDEs treatment, we examined TLR2 expression by immunofluorescent staining. The results showed the expression of TLR2 on astrocytes was increased after SCI, and significantly more robust after SCDEs treatment (Fig. 2a–c). Furthermore, it is well established that an upregulation of CSPGs within the glial scar and perineuronal net creates a barrier to axonal regrowth and sprouting [37]. By staining with CS56 to represent CSPGs, we found the deposition and area of CSPGs were decreased after SCDEs treatment relative to PBS group (Fig. 2d–f). Hence, we found that SCDEs treatment could upregulate the expression of TLR2 on astrocytes and attenuate CSPGs deposition in lesion area.

### SCDEs improved functional recovery after SCI in vivo

**Fig. 3:**
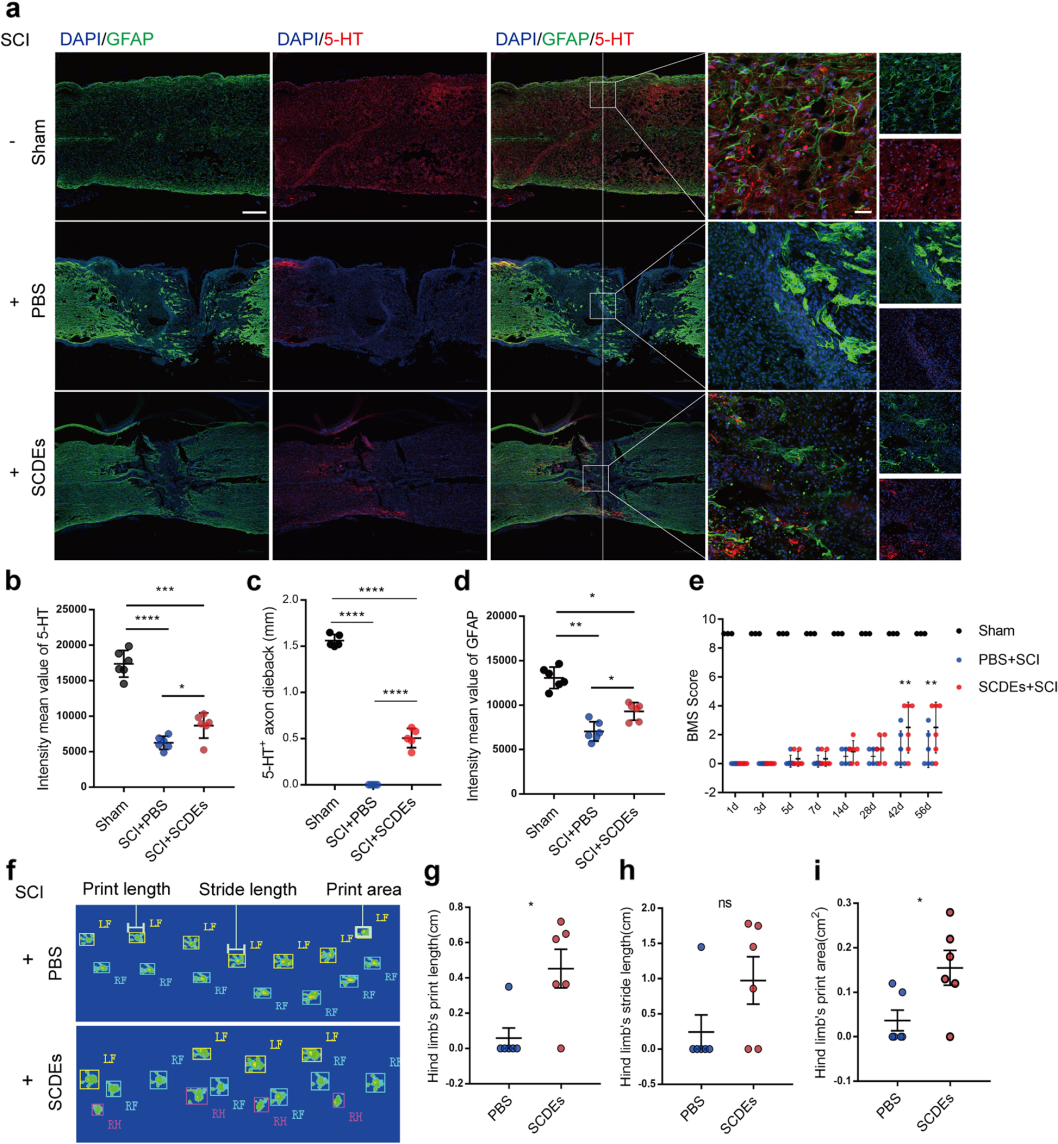
SCDEs improved functional recovery after SCI in vivo. **a** Representative images of immunofluorescent analysis of neurotransmitter marker 5-HT (red), GFAP (green), and DAPI (blue) in each group at 4 weeks after SCI. White arrow showed the downstream distance of 5-HT^+^ axons. Scale bars = 200 μm. Right images are high-resolution versions of the boxed regions in the left images, scale bars = 50 µm. **b**–**d** Quantitative analysis of 5-HT^+^ axon dieback (mm) and the intensity mean value of 5-HT and GFAP (*P < 0.05, **P < 0.01, ***P < 0.001, ****P < 0.0001, *n* = 6). **e** Quantitative analysis of BMS score between each group (**P < 0.01, *n* = 6). **f**–**i** Quantitative analysis of catwalk at 8 weeks, including print length, stride length, and print area (*P < 0.05, ns: no significance, *n* = 6, LF: left front paw, RF: right front paw, RH: right hind paw)

To determine the effects of SCDEs on the potential for neurological function recovery after SCI, we examined the expression of 5-HT and GFAP at 4 weeks post-SCI by immunofluorescent staining. The results showed robustly increased GFAP level and markedly more neurotransmitters have been transmitted downstream after SCDEs injected relative to PBS group (Fig. 3a–d). To further investigate the contribution of SCDEs on locomotive functional recovery, we performed BMS to demonstrate that the motor function recovery with SCDEs group was significantly greater than that achieved with PBS group (Fig. 3e). Gait analyses were performed using the Catwalk Automated Gait Analysis System, which showed SCDEs group mice had better recovery than PBS group mice on quantitative information about stride length, print length, and print area of hind limb (Fig. 3f). Thus, SCDEs could increase astrocytes expression and improve both neurological functional and locomotive functional recovery of mice after SCI.

### Astrocyte-specific TLR2 deficiency decreased the neuroprotective effect of SCDEs after SCI

**Fig. 4:**
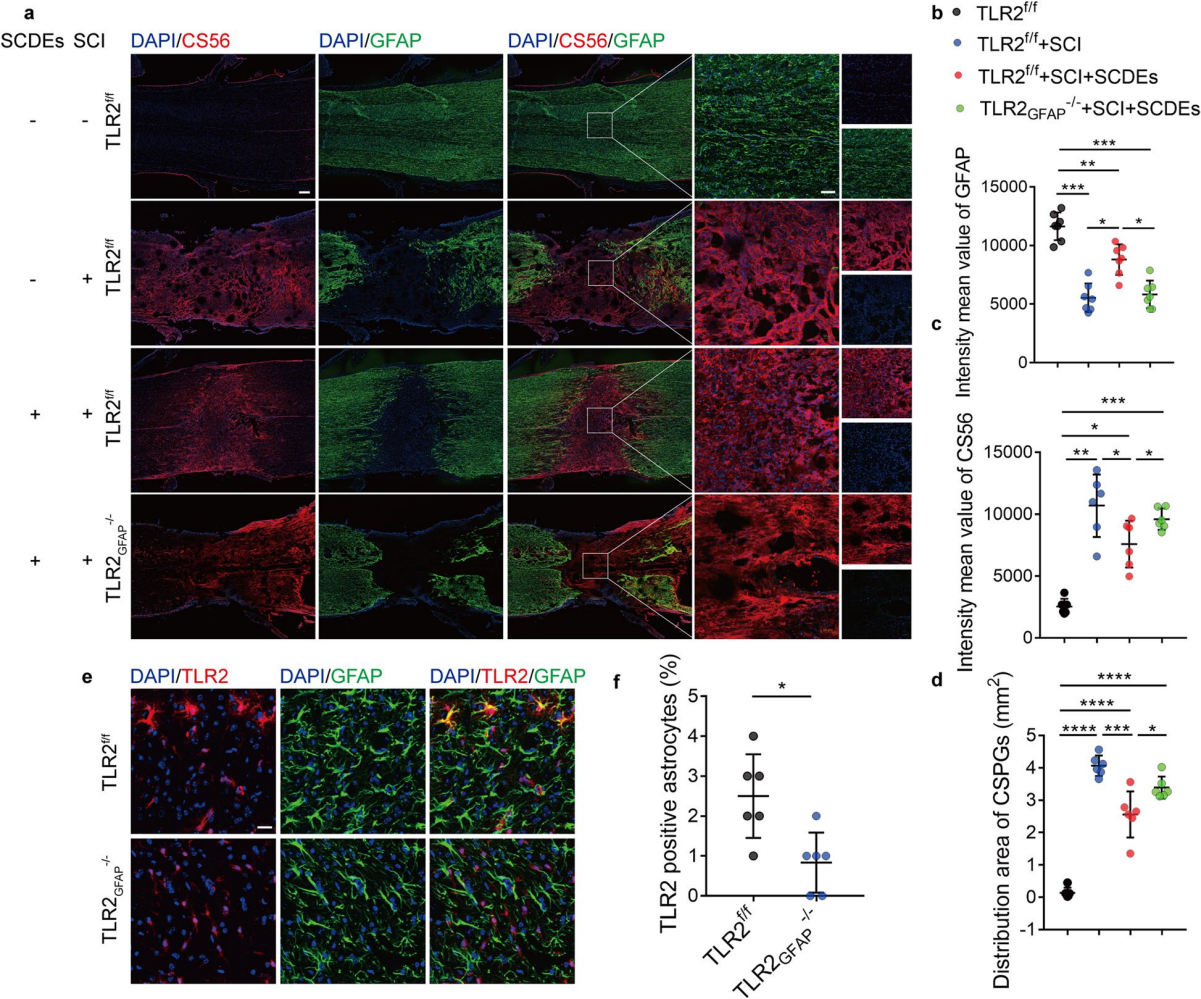
TLR2 deficiency on astrocytes abolished the decreased CSPGs deposition and upregulated astrocytes induced by SCDEs treatment. **a** Representative images of immunofluorescent analysis of CS56 (red), GFAP (green), and DAPI (blue) in TLR2^f/f^ sham group, TLR2^f/f^ group, TLR2^f/f^ with SCDEs treatment group and TLR2^−/−^group at 2 weeks after SCI. Scale bars = 200 μm. Right images are high-resolution versions of the boxed regions in the left images, scale bars = 50 µm. **b**–**d** Quantitative analysis of CSPGs area and the intensity value of CS56 and GFAP (*P < 0.05, **P < 0.01, ***P < 0.001, ****P < 0.0001, *n* = 6). **e** Representative images of immunofluorescent analysis of TLR2 (red), GFAP (green), and DAPI (blue) in TLR2^f/f^ and TLR2^−/−^ mice at 2 weeks after SCI. Scale bars = 20 μm. **f** Quantitative analysis of TLR2-positive astrocytes **P < 0.01, *n* = 6)

**Fig. 5:**
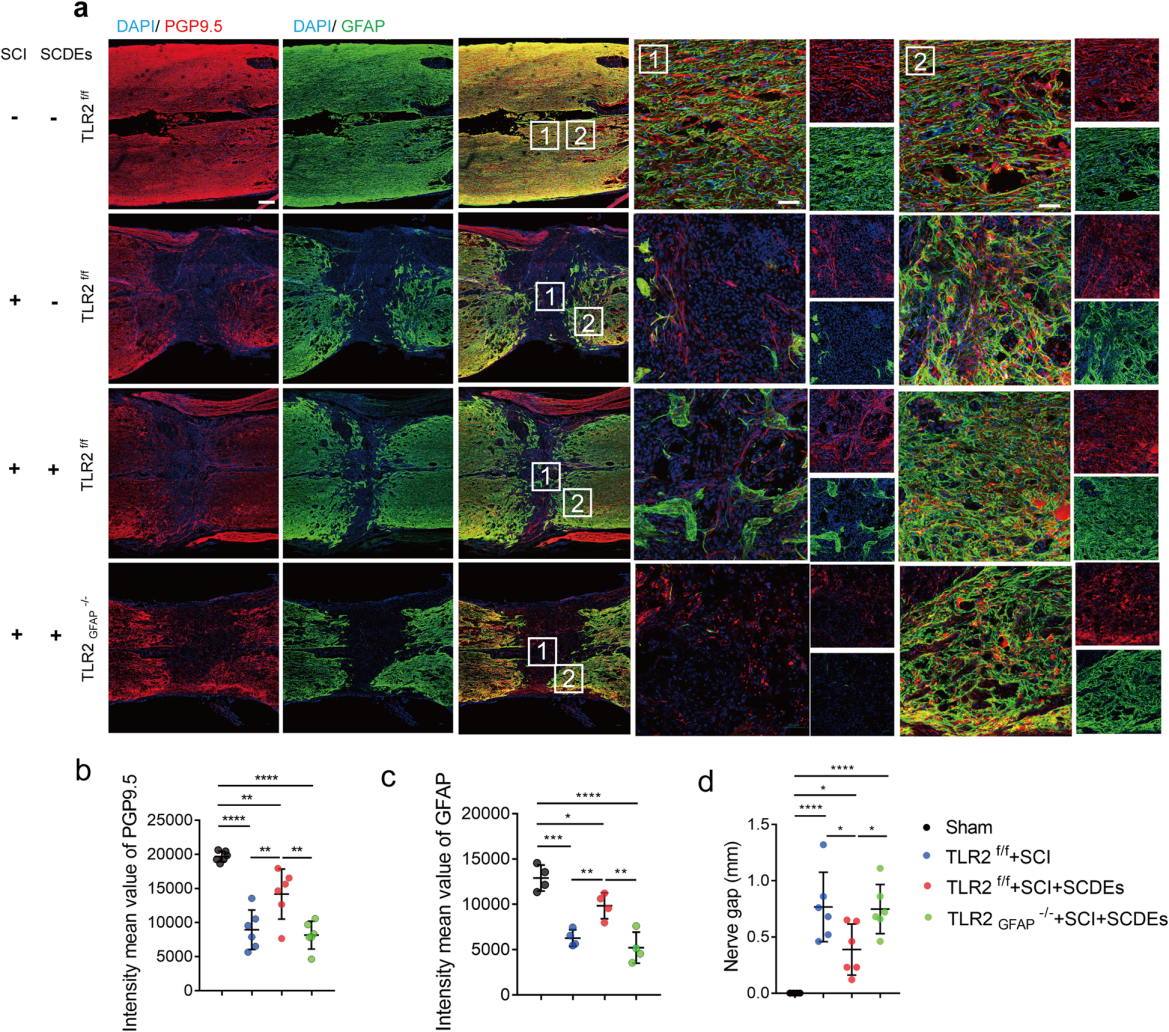
Astrocyte specific TLR2 deficiency decreased the neuroprotective effect of SCDEs after SCI. **a** Representative images of immunofluorescent analysis of nerve marker PGP9.5 (red), GFAP (green), and DAPI (blue) in each group at 2 weeks after SCI. Right 4 column images are high-resolution versions of the boxed regions in the third column images. Scale bars: left 3 column images = 200 μm. Right 4 column images = 50 μm. **b**–**d** Quantitative analysis of nerve gap and the intensity value of PGP9.5 and GFAP (*P < 0.05, **P < 0.01, ***P < 0.001, ****P < 0.0001, *n* = 6)

To further validate our hypothesis that SCDEs could improve functional recovery after SCI via upregulated expression of TLR2. TLR2^f/f^ mice were crossed to the GFAP-Cre strain to generate astrocyte-specific TLR2 knockout mice (TLR2^−/−^). The knockout efficiency was validated by staining TLR2 in TLR2^−/−^ mice and TLR2^f/f^ mice (Fig. 4e, f). The results indicated the release of CSPGs and the area of CSPGs distribution were markedly decreased after SCDEs treatment. However, CSPGs deposition increased again in TLR2^−/−^ mice even with the SCDEs treatment (Fig. 4a–d). Furthermore, after SCDEs treatment, highly improved neurological function recovery with narrowed nerve gap, higher nerve distribute density, and increased astrocytes distribution were detected after SCDEs treatment by staining PGP9.5 and GFAP (Fig. 5a–d). However, TLR2^−/−^ mice abolished the improved nerve function recovery and decreased the density of reactive astrocytes in the lesion site (Fig. 5a–d). Collectively, these results indicate that SCDEs could improve functional recovery after SCI by enhancing the expression of TLR2, however, delete TLR2 in the astrocytes could eliminate the improvements induced by SCDEs and significantly upregulated the deposition of CSPGs in the lesion site after SCI.

### Upregulated TLR2 expression on astrocytes induced by SCDEs was via NF-κB/PI3K signaling pathway

**Fig. 6:**
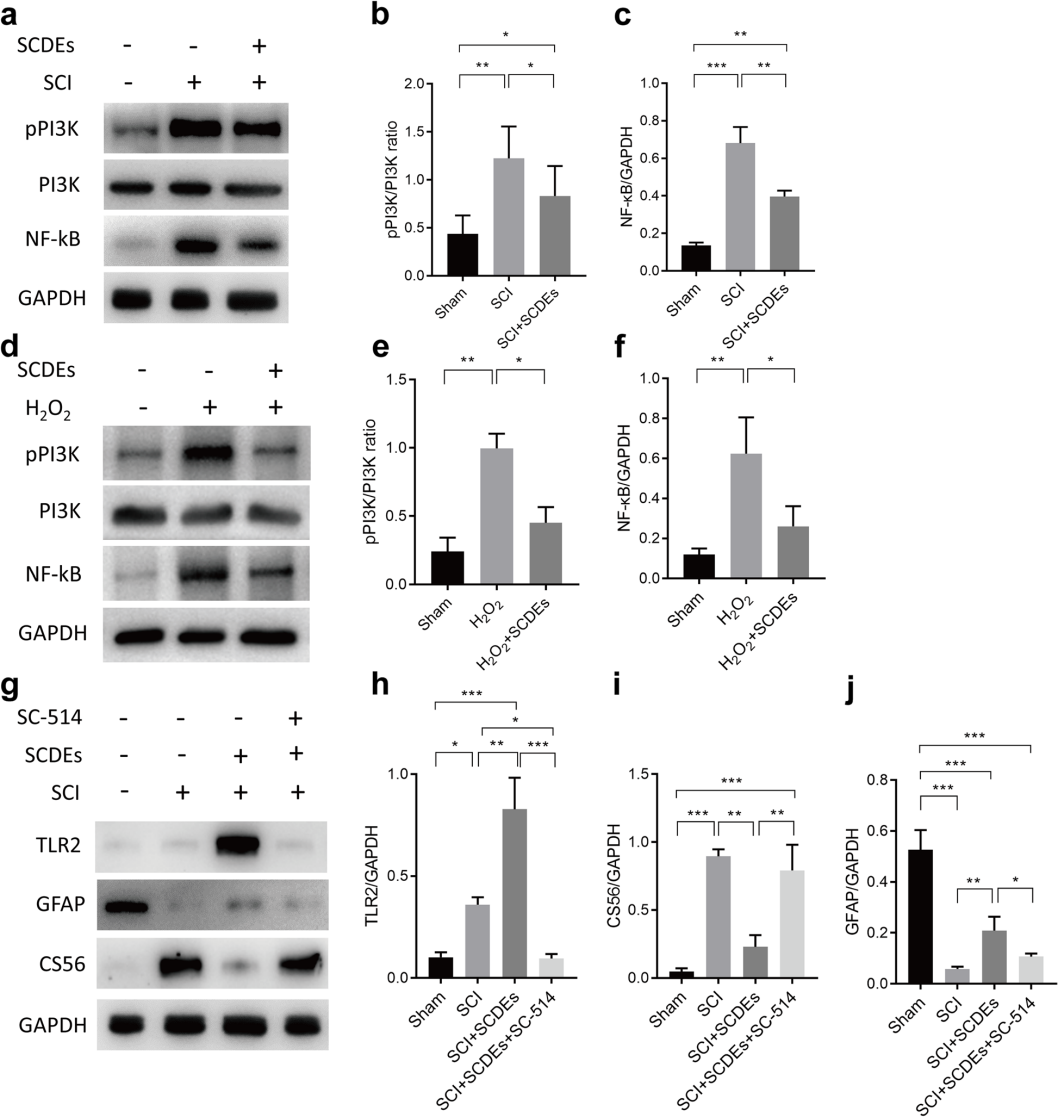
Upregulated TLR2 expression on astrocytes induced by SCDEs was via NF-κB/PI3K signaling pathway. **a** Representative western blots showing the activation of NF-κB/PI3K signaling in vivo. **b**, **c** Quantitative analysis of the pPI3K/PI3K ratio and NF-κB/GAPDH ratio in PBS group mice after SCI, SCDEs group mice after SCI, and control mice without surgery (sham). **d** Representative western blots showing the activation of NF-κB/PI3K signaling in vitro. **e**, **f** Quantitative analysis of the pPI3K/PI3K ratio and NF-κB/GAPDH ratio in sham group astrocytes, PBS group astrocytes with H_2_O_2_ incubation, and SCDEs treated astrocytes with H_2_O_2_ incubation. **g** Representative western blots validating the activation of NF-κB/PI3K signaling. **h**–**j** Quantitative analysis of the TLR2/GAPDH, CS56/GAPDH, and GFAP/GAPDH ratio in PBS group mice after SCI, SCDEs group mice after SCI, SCDEs and SC-514 group mice after SCI, and control mice without surgery (sham). (*P < 0.05, **P < 0.01, ***P < 0.001, ****P < 0.0001, *n* = 3)

To understand the detailed molecular mechanism and determine the importance of NF-κB dependent signaling pathways in regulating astrocytic TLR2 expression in vivo and in vitro, western blots were performed. The results indicated that the NF-κB/PI3K pathway played a critical role in decreasing TLR2 expression on astrocytes (Fig. 6a–f). In parallel, to validate the NF-κB/PI3K pathway was capable of regulating the TLR2 expression, astrocytes number and CSPGs releasing. Western blots were performed. The results showed that the IKK inhibitors SC-514 (ab144415, Abcam), respectively, was capable of attenuating the TLR2 levels in response to SCDEs, decreasing astrocytes distribution and enhancing CSPGs releasing (Fig. 6g–j). Together, upregulated TLR2 expression on astrocytes, which was induced by SCDEs, was triggered by NF-κB/PI3K signaling pathway.

### SC-514 abolished the effect of SCDEs on astrocytes distribution, TLR2 expression and release of CSPGs

**Fig. 7:**
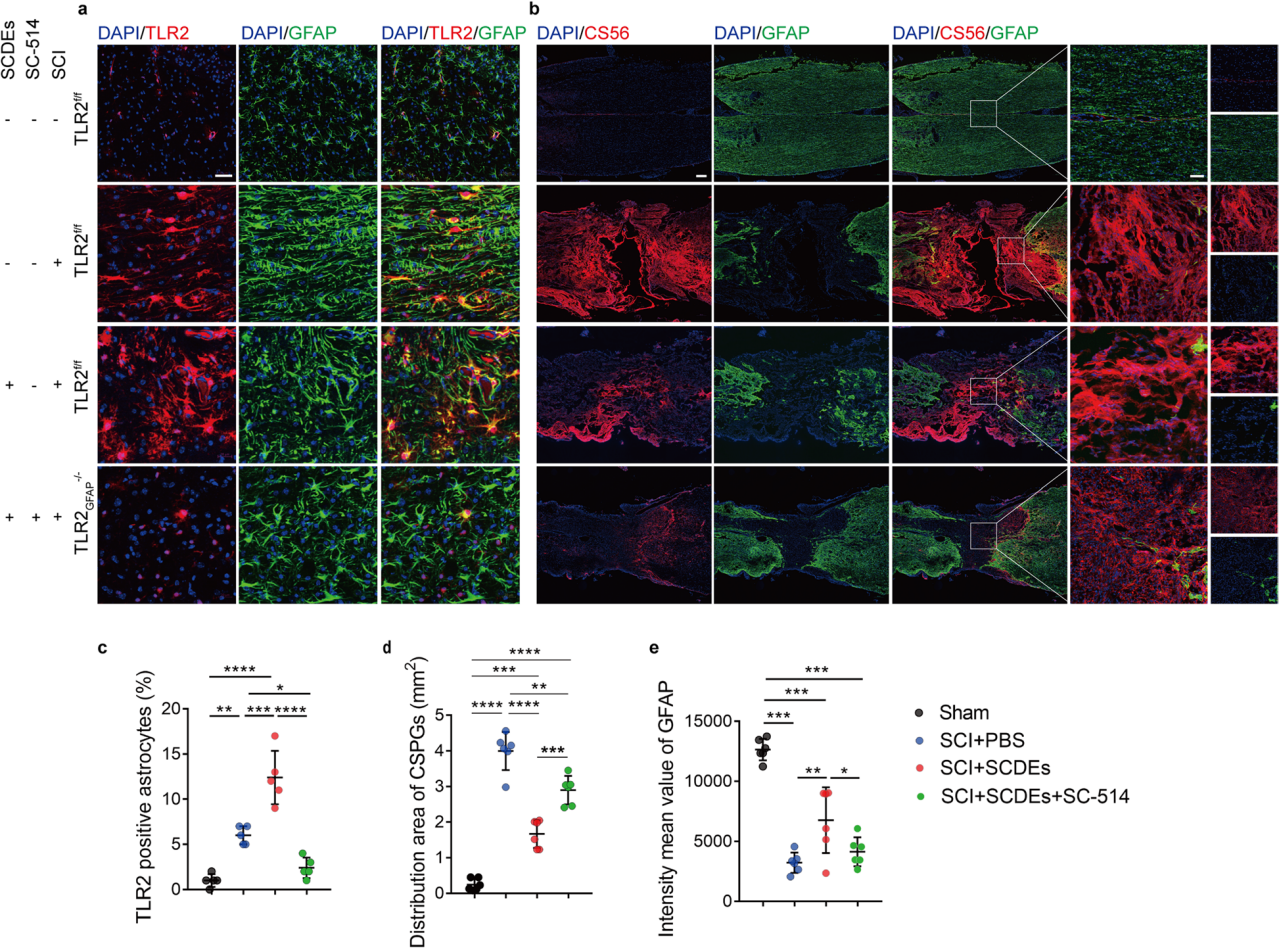
SC-514 abolished the effect of SCDEs on astrocytes distribution, TLR2 expression and release of CSPGs. **a** Representative images of immunofluorescent analysis of TLR2 (red), GFAP (green) and DAPI (blue) in each group at 2 weeks after SCI. Scale bars = 50 μm. **b** Representative images of immunofluorescent analysis of CS56 (red), GFAP (green), and DAPI (blue) in each group at 2 weeks after SCI. Scale bars = 200 μm. Right images are high-resolution versions of the boxed regions in the left images, scale bars = 50 µm. **c**–**e** Quantitative analysis of TLR2-positive astrocytes, CSPGs area and the intensity value of GFAP (*P < 0.05, **P < 0.01, ***P < 0.001, ****P < 0.0001, *n* ≥ 5)

To validate the NF-κB/PI3K signaling pathway in vivo, mice with intraperitoneal injection (i.p) of SC-514 twice a week along with tail vein injection of SCDEs after SCI were defined to be the SC-514 and SCDEs group. Mice injected with SCDEs after SCI were defined as SCDEs group. Control group mice were injected with PBS after SCI and no clip surgery on spinal cord in sham group mice. The results revealed that SC-514 could markedly abolish the upregulated TLR2 expression on astrocytes, increased astrocytes distribution, and decreased CSPGs releasing caused by SCDEs injection (Fig. 7a–d). Taken together, our data further confirmed that the NF-κB/PI3K signaling pathway was the critical trigger in the expression of TLR2 on astrocytes caused by SCDEs treatment, thereby leading to decreased CSPGs deposition in the lesion site to promote axon growth and neuron survival after spinal cord injury.

## Discussion

**Fig. 8:**
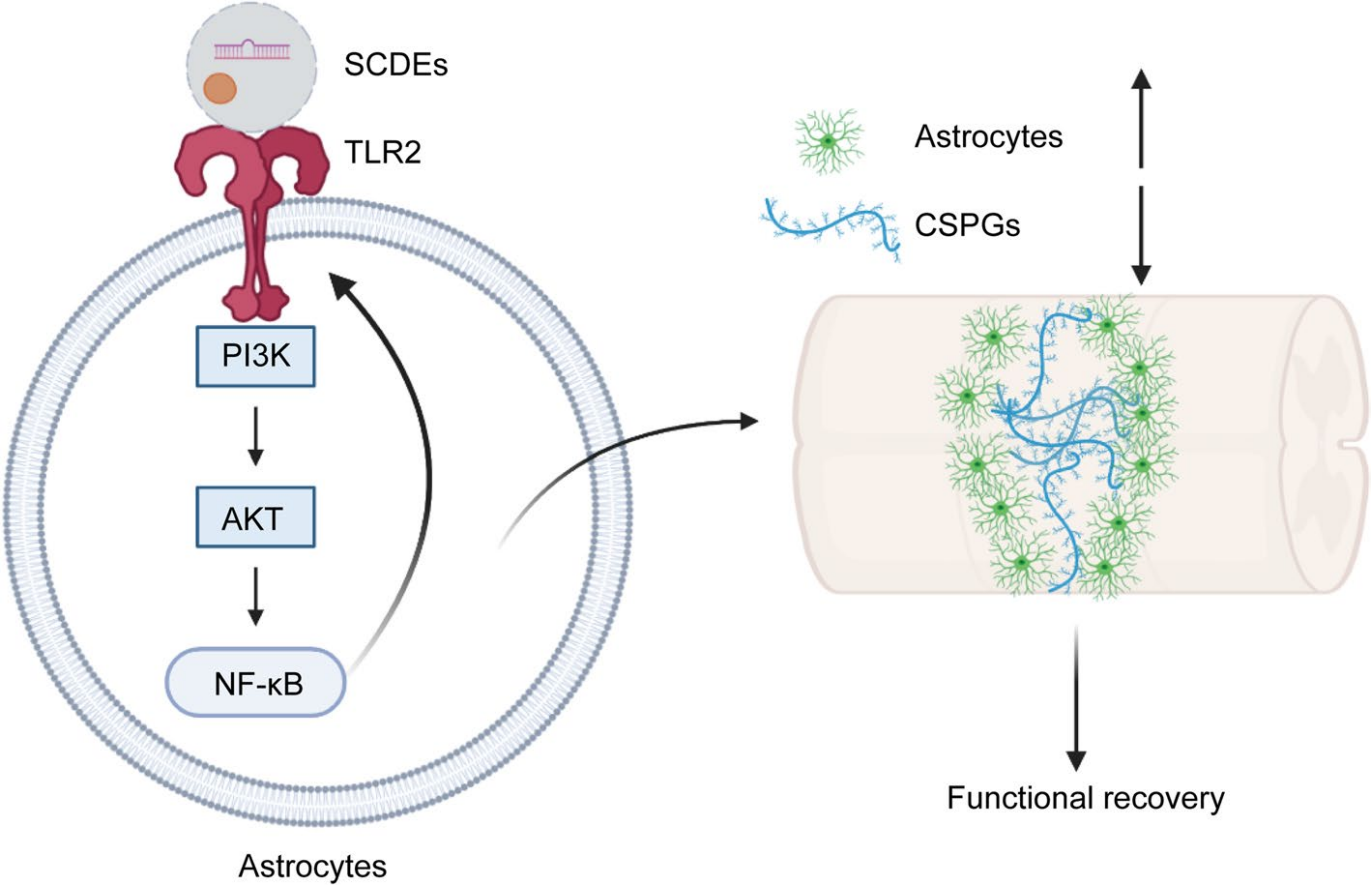
Schematic diagram showing the findings of this study

The present study shows, for the first time, that the treatment of SCDEs could notably stimulate the TLR2 expression on astrocytes and reduce the CSPGs secretion post-SCI. Moreover, KO of TLR2 on astrocytes can reverse the change caused by SCDEs treatment. Decreased CSPGs deposition after SCDEs treatment, which was led by induced TLR2 expression on astrocytes, was triggered by NF-κB/PI3K signaling pathway and IKKβ inhibitor SC-514 was also used to validate this signaling pathway. These findings suggest that SCDEs treatment may play a critical role in the functional recovery after SCI by activating TLR2 on astrocytes (Fig. 8).

SCI is a severely disabling disease that leads to loss of sensation, motor, and autonomic function [38]. Exosomes are endogenous nanovesicles and play a key role in the signal transmission between cells. Exosomes have great potential in diagnosis, prognosis, and treatment because of their ability to easily cross the blood–brain barrier and be able to deliver molecules specifically to the CNS [39]. SCs are the myelinating glia of the peripheral nervous system and they guide regenerating axons following peripheral nerve injury [40]. In addition, SCs transplantation has been comprehensively studied as a strategy for SCI repair [41]. However, far too few studies have focused on SCDEs.

TLRs are a class of proteins involved in the immune system [42]. Only a few TLRs, such as TLR2, TLR4, and TLR9, have been studied in SCI [43]. TLR2 is widely investigated in the pathogenesis of autoimmune diseases including rheumatoid arthritis [44–46], systemic lupus erythematosus [47, 48], systemic sclerosis [49], Sjogren’s syndrome [50–52], psoriasis [53, 54], multiple sclerosis [55, 56], and autoimmune diabetes [57, 58]. After SCI, increased TLR2 expressed on reactive astrocytes and no significant changes of TLR2 expression on native astrocytes (Supplementary Figure 4). Except reactive astrocytes, which were the mainly CSPGs produced cells, oligodendrocytes and microglia could also produce CSPGs [7, 43, 59]. We did a quantitative analysis of the proportion of CSPGs deposited in astrocytes area and areas without astrocytes to the total deposited area (Supplementary Figure 2). The results showed the ratio of CSPGs deposited in area with astrocytes to whole CSPGs deposited area was around 50–60%, and no difference between PBS group and SCDEs group. In addition, some CSPGs were deposited in the center of the injury (less than 50%), which we believe were produced by other cells. Some studies have shown activation of microglia TLR2 has neuroprotective effects post-SCI and TLR4 can also play beneficial roles in SCI [60, 61]. Although TLR2 is shown to contribute to the nerve injury-induced spinal cord glial cell activation [62], the mechanism of axon regeneration and neurons survival mediated by TLR2 is still unclear.

Following SCI, CSPGs are rich in lesion area and associated with specialized structures termed perineuronal nets (PNNs) which surround the soma and dendrites of mature neurons [63]. There are also different kinds of CSPGs binding to the hyaluronan backbone of the PNN, like aggrecan, neurocan, brevican, phosphacan, and versican [64]. CSPGs depositions are the main inhibitory factors associated with axon growth and neurons survival [65–67]. However, the studies about one of the CSPGs secreting cells, astrocytes, were really controversial. Contrary to the widely accepted inhibitory function of glia scar by inhibit axon regeneration, many studies have shown the glia scar forming cells, astrocytes, have beneficial effect on neuronal development, synapse formation and proper propagation of action potentials [68, 69]. In our study, we found improved functional recovery after SCDEs treatment comes along with increased astrocytes and a recent study characterizing the varying phenotypes of astrocytes can partially explain this controversial problem. And using a fluorescent tag or antibody labeling to trace the SCDEs to the lesion site was our next goal to reveal the delivery. In this study, astrocytes related to glia scar can be divided into three subtypes: the naïve astrocytes, the reactive astrocytes, and the scar-forming astrocytes. The inhibitory glia scar may most consist of scar-forming astrocytes [70].

In summary, we indicate that SCDEs can promote functional recovery of mice post-SCI by decreasing the CSPGs deposition via upregulating the TLR2 expression on astrocytes through NF-κB/PI3K signaling pathway. The present study provides potential therapeutic way for the treatment of SCI.

## Conclusion

Our results uncovered that SCDEs can promote functional recovery of mice post-SCI by decreasing the CSPGs deposition via increasing the TLR2 expression on astrocytes through NF-κB/PI3K signaling pathway.

## Supplementary Information

**Additional file 1: Supplementary Figure 1.** Characterization of isolated Schwann cell. (a) Representative images of immunofluorescent analysis of Schwann cell marker S100 (red), p75 (green) and DAPI (blue) Scale bars= 75 μm.

**Additional file 2: Supplementary Figure 2.** (a) Quantitative analysis of CSPGs deposited in area with astrocytes/ whole CSPGs deposited area (%).

**Additional file 3: Supplementary Figure 3.** Characterization of isolated astrocytes. (a) Representative images of immunofluorescent analysis of astrocytes marker GFAP (green) and DAPI (blue) Scale bars= 200 μm.

**Additional file 4: Supplementary Figure 4.** (a). Representative images of immunofluorescent analysis of astrocytes marker GFAP (green), CS56 (red) and DAPI (blue) Scale bars= 50 μm. (b). Quantitative analysis of the intensity mean value of CS56^+^ area (**P<0.01, *n*=4). (c). Representative western blots showing the expression of CS56 *in vivo*. (d). Quantitative analysis of the CS56/GAPDH ratio in SCI group and control mice without surgery (Sham). (**P<0.01, *n*=3).

**Additional file 5: Supplementary Figure 5.** (a).TLR2 Expression difference native astrocytes and reactive astrocytes between PBS and SCDEs treatment after SCI. (a) Representative images of immunofluorescent analysis of astrocytes marker GFAP (green), TLR2 (red) and DAPI (blue) Scale bars= 50 μm. (b). Schematic pattern of selected region of native astrocyte and reactive astrocyte after SCI. (c, d). Quantitative analysis of the TLR2 positive native astrocytes and reactive astrocytes in the selected region (*P<0.05, N.S: no significance, *n*=5).

**Additional file 6: Supplementary Figure 6. **(a). Representative western blots showing the expression of TLR2 and GFAP *in vivo*. (b, c). Quantitative analysis of the TLR2/GAPDH ratio and GFAP/GAPDH ration in mice after SCI with or without SCDEs treatment. (*P<0.05, *n*=3).

**Additional file 7: Supplementary Figure 7.** (a). Decreased fibrotic scar formation after SCDEs treatment. (a) Representative images of immunofluorescent analysis of fibrotic scar marker fibronectin (green), Collagen III (red) and DAPI (blue) Scale bars= 200 μm. (b, c). Quantitative analysis of the intensity mean value of fibronectin and collagen III positive area (*P<0.05, ** P<0.01, *** P<0.001, *n*=4).

## Data Availability

Not applicable.

## Abbreviations

SCI: Spinal cord injury
SCDEs: Schwann cell-derived exosomes
TLR2: Toll-like receptor 2
CSPGs: Chondroitin sulfate proteoglycans
PNS: Peripheral nervous system
CNS: Central nervous system
KO: Knockout
WT: Wild type
TLR2^−/−^: GFAP-Cre; TLR2^flox/flox^
TLR2^f/f^: TLR2 flox allele
i.p.: Intraperitoneal
FBS: Fetal bovine serum
PBS: Phosphate-buffered saline
DPBS: Dulbecco’s phosphate-buffered saline
RIPA: Radioimmunoprecipitation assay
PVDF: Polyvinylidene difluoride
BSA: Bovine serum albumin
GAPDH: Glyceraldehyde 3-phosphate dehydrogenase
SD: Standard deviations
ANOVA: Analysis of variance
DAPI: 4′,6-Diamidino-2-phenylindole
TEM: Transmission electron microscopy
PNNs: Perineuronal nets
