# Supplementary figures and images for: Increasing toll-like receptor 2 on astrocytes induced by Schwann cell-derived exosomes promotes recovery by inhibiting CSPGs deposition after spinal cord injury
Source: J Neuroinflammation. 2021 Aug 9;18:172. doi: 10.1186/s12974-021-02215-x (PMC8353762; doi:10.1186/s12974-021-02215-x)

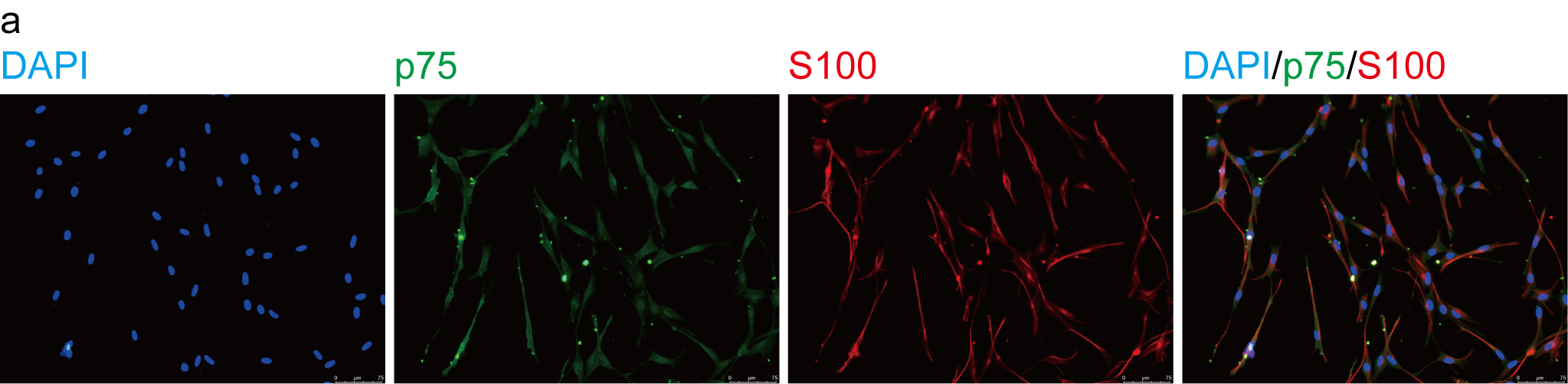

Supplement: Supplementary file 1 — Additional file 1: Supplementary Figure 1. Characterization of isolated Schwann cell. (a) Representative images of immunofluorescent analysis of Schwann cell marker S100 (red), p75 (green) and DAPI (blue) Scale bars= 75 μm. [file 12974_2021_2215_MOESM1_ESM.tif]

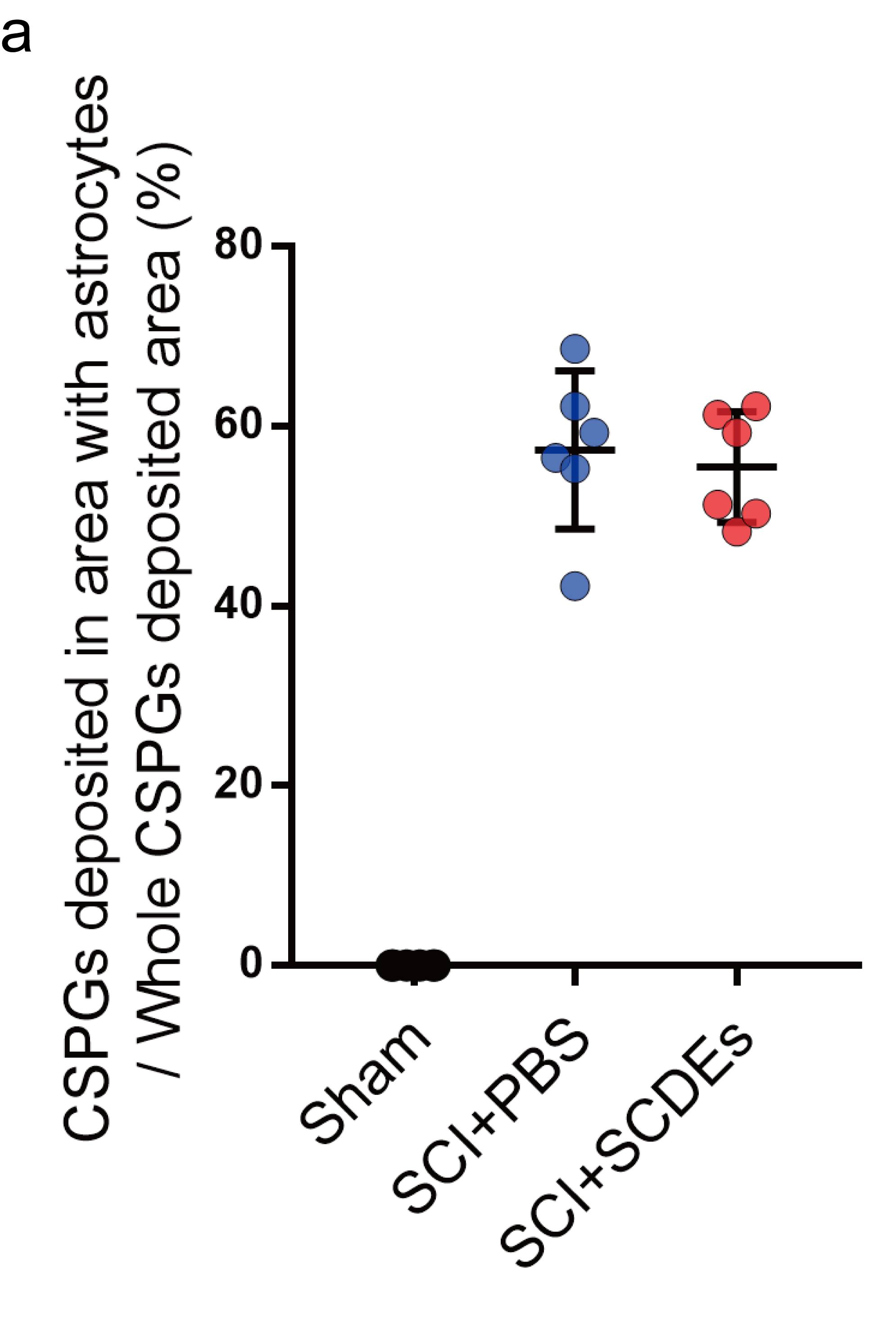

Supplement: Supplementary file 2 — Additional file 2: Supplementary Figure 2. (a) Quantitative analysis of CSPGs deposited in area with astrocytes/ whole CSPGs deposited area (%). [file 12974_2021_2215_MOESM2_ESM.tif]

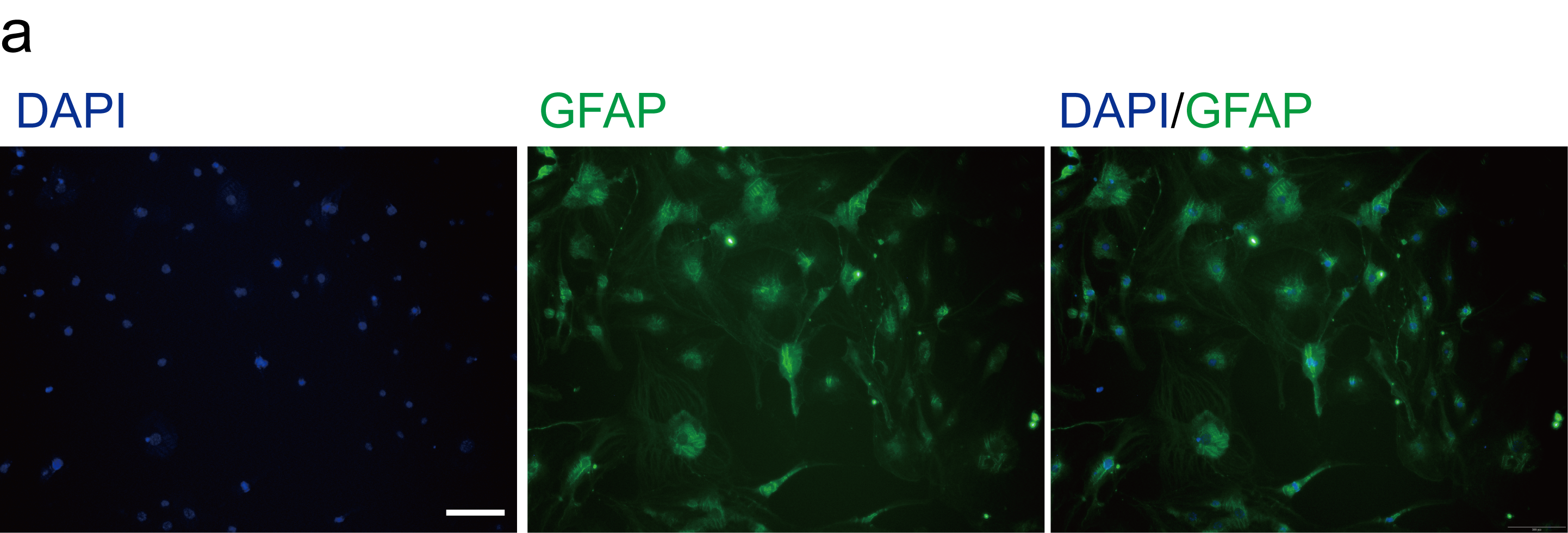

Supplement: Supplementary file 3 — Additional file 3: Supplementary Figure 3. Characterization of isolated astrocytes. (a) Representative images of immunofluorescent analysis of astrocytes marker GFAP (green) and DAPI (blue) Scale bars= 200 μm. [file 12974_2021_2215_MOESM3_ESM.tif]

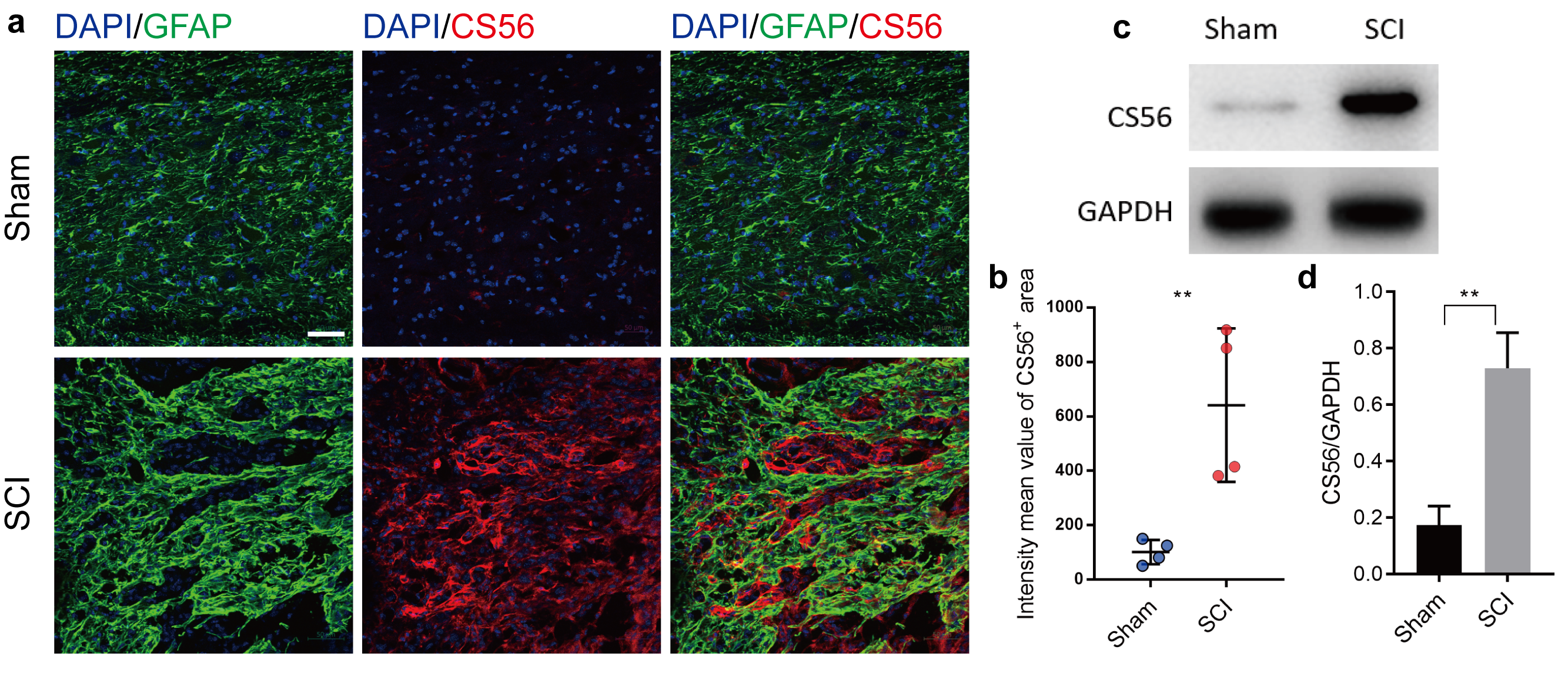

Supplement: Supplementary file 4 — Additional file 4: Supplementary Figure 4. (a). Representative images of immunofluorescent analysis of astrocytes marker GFAP (green), CS56 (red) and DAPI (blue) Scale bars= 50 μm. (b). Quantitative analysis of the intensity mean value of CS56+ area (**P<0.01, n=4). (c). Representative western blots showing the expression of CS56 in vivo. (d). Quantitative analysis of the CS56/GAPDH ratio in SCI group and control mice without surgery (Sham). (**P<0.01, n=3). [file 12974_2021_2215_MOESM4_ESM.tif]

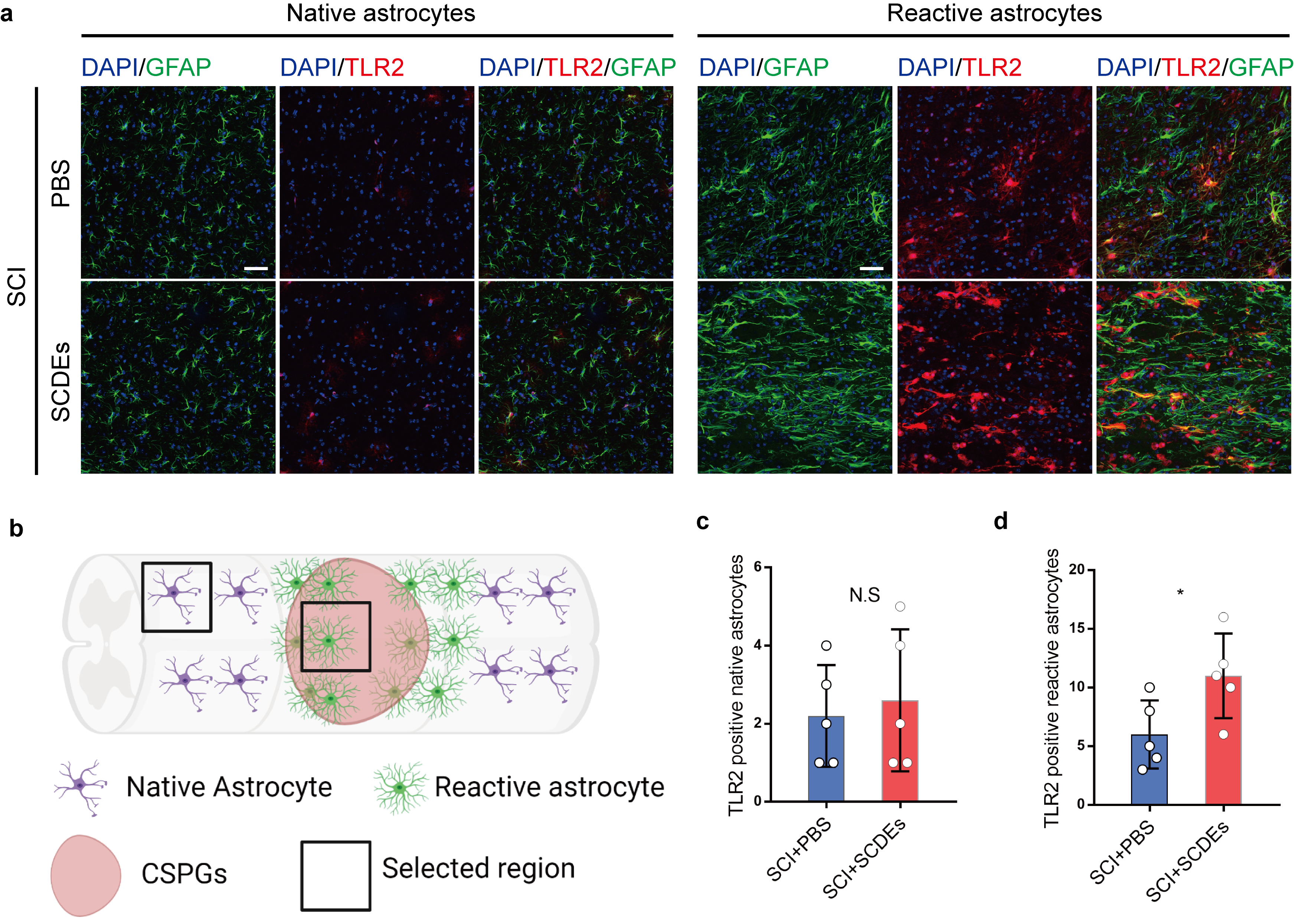

Supplement: Supplementary file 5 — Additional file 5: Supplementary Figure 5. (a).TLR2 Expression difference native astrocytes and reactive astrocytes between PBS and SCDEs treatment after SCI. (a) Representative images of immunofluorescent analysis of astrocytes marker GFAP (green), TLR2 (red) and DAPI (blue) Scale bars= 50 μm. (b). Schematic pattern of selected region of native astrocyte and reactive astrocyte after SCI. (c, d). Quantitative analysis of the TLR2 positive native astrocytes and reactive astrocytes in the selected region (*P<0.05, N.S: no significance, n=5). [file 12974_2021_2215_MOESM5_ESM.tif]

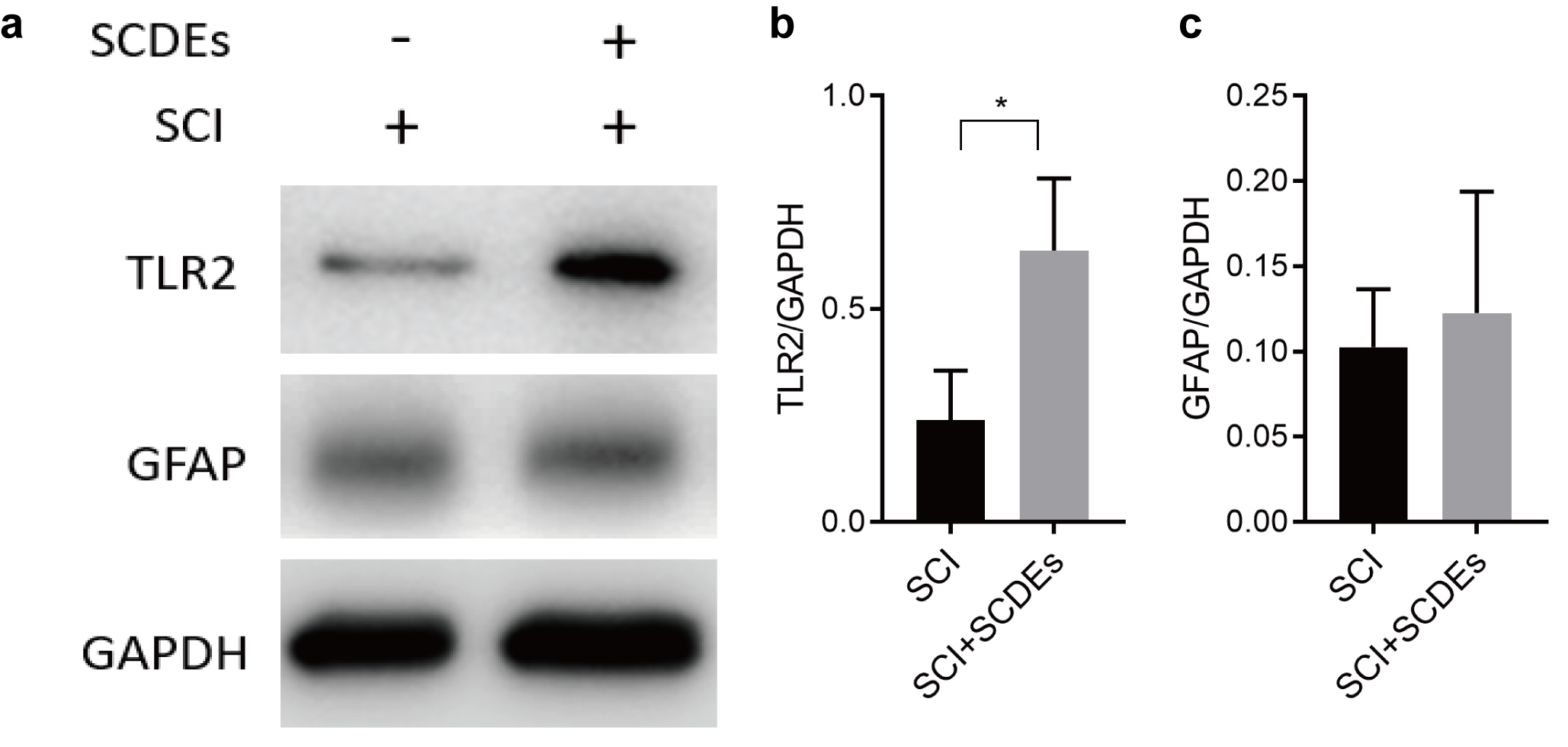

Supplement: Supplementary file 6 — Additional file 6: Supplementary Figure 6. (a). Representative western blots showing the expression of TLR2 and GFAP in vivo. (b, c). Quantitative analysis of the TLR2/GAPDH ratio and GFAP/GAPDH ration in mice after SCI with or without SCDEs treatment. (*P<0.05, n=3). [file 12974_2021_2215_MOESM6_ESM.tif]

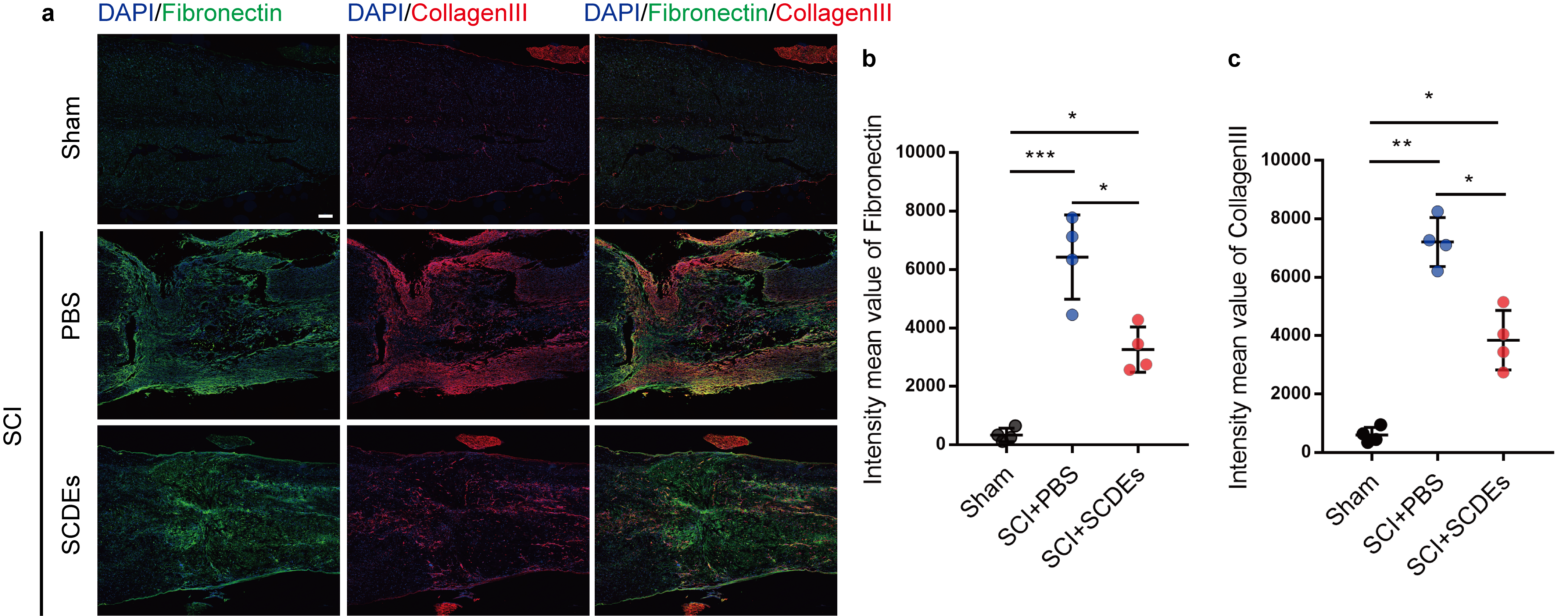

Supplement: Supplementary file 7 — Additional file 7: Supplementary Figure 7. (a). Decreased fibrotic scar formation after SCDEs treatment. (a) Representative images of immunofluorescent analysis of fibrotic scar marker fibronectin (green), Collagen III (red) and DAPI (blue) Scale bars= 200 μm. (b, c). Quantitative analysis of the intensity mean value of fibronectin and collagen III positive area (*P<0.05, ** P<0.01, *** P<0.001, n=4). [file 12974_2021_2215_MOESM7_ESM.tif]
